# Totally endoscopic systemic atrioventricular valve re-replacement for congenitally corrected transposition of the great arteries: case report

**DOI:** 10.1093/ehjcr/ytaf340

**Published:** 2025-07-14

**Authors:** Riku Kato, Toshiaki Ito

**Affiliations:** Department of Cardiovascular Surgery, Japanese Red Cross Aichi Medical Center Nagoya Daiichi Hospital, Michishita, Nagoya 453-8511, Japan; Department of Cardiovascular Surgery, Japanese Red Cross Aichi Medical Center Nagoya Daiichi Hospital, Michishita, Nagoya 453-8511, Japan

**Keywords:** Totally endoscopic, Minimally invasive cardiac surgery, Congenitally corrected transposition of the great arteries, Systemic atrioventricular valve, Case report

## Abstract

**Background:**

Congenitally corrected transposition of the great arteries (ccTGA) is often associated with systemic atrioventricular valve (SAVV) regurgitation and the dysfunction of the systemic ventricle.

**Case summary:**

We describe a case of a 47-year-old female who underwent SAVV replacement with bioprosthetic valve at 39-year-old through left thoracotomy approach. She developed dyspnoea on exertion and was recommended for surgery. We performed a totally three-dimensional endoscopic minimally invasive re-replacement of SAVV.

**Discussion:**

Endoscopic replacement of SAVV for an adult is possible with an excellent view. We describe our approach which has overcome unique technical hurdles and has yielded favourable outcomes.

Learning points

- It’s crucial to perform valve replacement in adult congenitally corrected transposition of the great arteries (ccTGA) patients before the right ventricular function deteriorates due to valve regurgitation.
- Various surgical approaches, including left thoracotomy and right chest endoscopic methods, offer effective alternatives for accessing the SAVV in ccTGA patients, accommodating their unique anatomical challenges.

## Introduction

Congenitally corrected transposition of the great arteries (ccTGA) is a rare cardiac malformation characterized by double discordance. Nearly 90% of ccTGA patients have complications with other congenital heart diseases, such as ventricular septal defects and pulmonary stenosis, often requiring anatomical repairs during the infancy.^1^ However, ccTGA patients without additional cardiac complications and symptoms may not require surgery in childhood. Many of these patients must undergo systemic atrioventricular valve (SAVV) replacement in adulthood due to SAVV regurgitation. The unique anatomy of ccTGA makes securing the visualization of SAVV challenging. Herein, we report a case where a totally 3D endoscopic approach via right thoracotomy provided a good visualization of the SAVV.

## Summary figure

**Figure:**
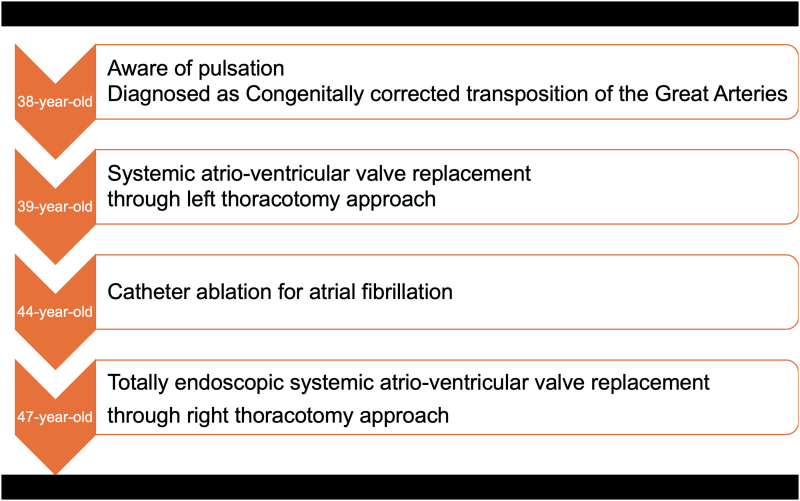


## Case report

**Figure 1:**
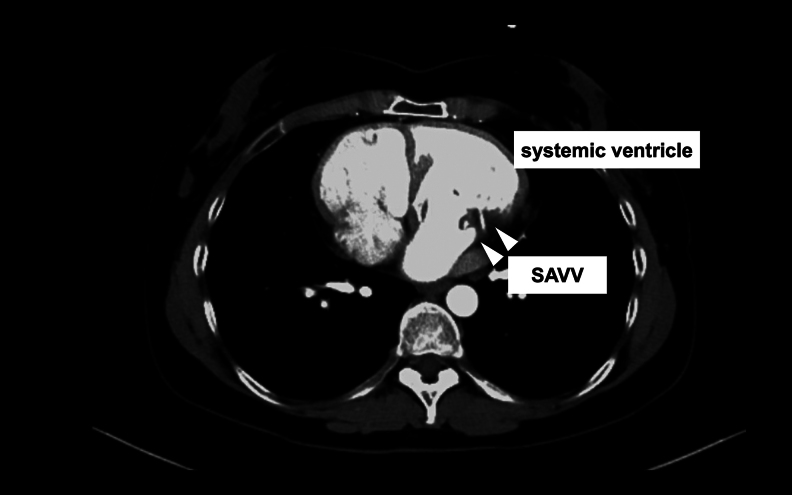
Contrast-enhanced computed tomography showed that systemic atrioventricular valve was vertically oriented (white arrow).

A 47-year-old female with ccTGA, who undergone SAVV replacement with a bio-prosthesis (Epic 27 mm; Abbott Laboratories, IL, USA) as a 39-year-old and catheter ablation for atrial fibrillation as a 44-year-old, presented with dyspnoea on exertion. Her transthoracic echocardiography showed that biventricular function remained normal (functional left ventricular ejection fraction: 65%, functional right ventricular ejection fraction: 55%) and the end-diastolic and end-systolic diameters of the functional left ventricle were 58 and 37 mm, respectively. The mean trans-prosthetic pressure gradient was 14 mmHg, and the degree of SAVV regurgitation was moderate (*Video 1*). Given her symptoms, she was referred to our institution for surgical re-replacement. The index surgery was performed via left thoracotomy and left atriotomy thorough the left atrial appendage at the prior institution to address the anatomical challenge of ccTGA. During that procedure, the right jugular vein was injured when inserting a venous drainage tube, resulting in a right haemothorax. A bioprosthetic valve had been selected due to her desire for future pregnancy. Her contrast-enhanced computed tomography scan illustrated that the SAVV was vertically oriented (*Figure 1*, *Video 2*), indicating that visualization through the conventional median sternotomy approach would be challenging. Moreover, she also strongly hoped to avoid median sternotomy. Based on our experience, we anticipated that the totally endoscopic approach through the right chest with a three-dimensional (3D) endoscope could provide a better surgical view of the SAVV.

**Figure 2:**
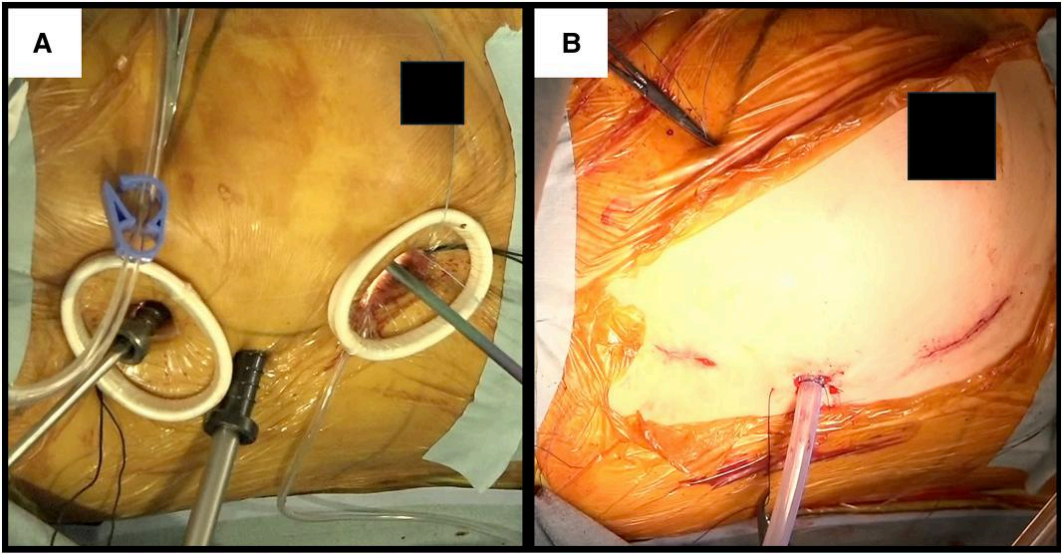
(*A*) Setup of intraoperative port setting, (*B*) postoperative surgical wound.

**Figure 3:**
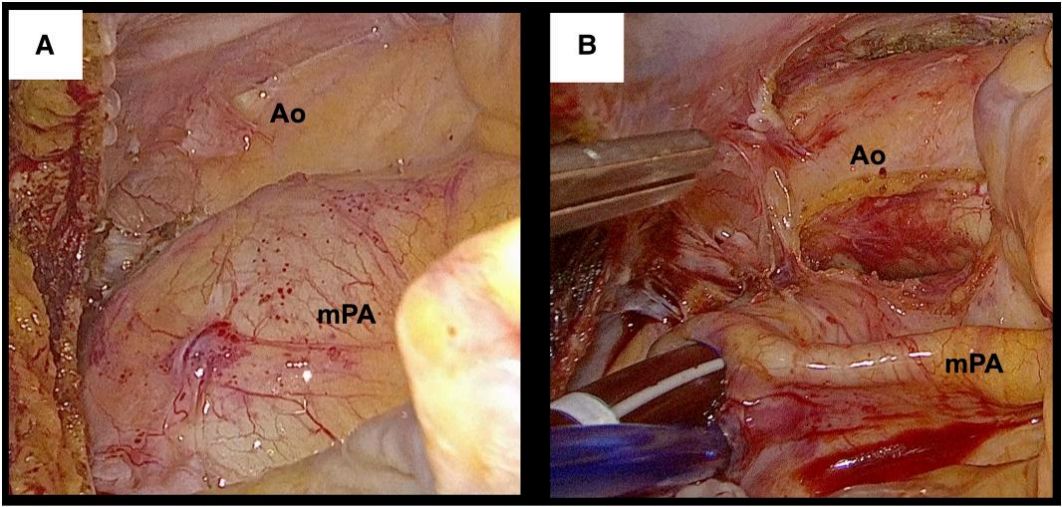
3D-endoscopic view before inserting vent tube into the main trunk of the pulmonary artery (*A*), and after inserting vent tube (*B*). Ao, aorta.

Under general anaesthesia, she was placed in a thirty-degree left lateral decubitus position with the right arm fixed over the head. Cardiopulmonary bypass (CPB) was established through the right groin. Distal perfusion of the right leg was performed using 6 Fr sheath because the right femoral artery was so small. A 10 mm trocar for a 3D endoscope (Karl Storz, Tuttlingen, Germany) was inserted through the fourth intercostal space. The main 4 cm incision was made at the fifth intercostal space without rib-spreading. A 2 cm incision for a left-hand instrument was placed at the third intercostal space (*Figure 2A* and *B*). The intrathoracic and intrapericardial adhesion were not severe, despite the previous haemothorax. Upon opening the pericardium, the dilated main trunk of the pulmonary artery (mPA) which was positioned to the right side of the ascending aorta prevented exposure of it (*Figure 3A*). Inserting a vent tube into mPA through the cranial-side window brought mPA collapsed, improving the visualization of the ascending aorta (*Figure 3C*). The ascending aorta was cross clamped using a flexible clamp, and cardiac arrest was achieved through antegrade cardioplegia. The vent tube, a cardioplegia needle, and the aortic clamp were inserted through the cranial-side window. Through a right-side left atriotomy, the sclerotic bioprosthetic valve was exposed with a similar view with usual endoscopic mitral valve surgeries. Although the valve was removed with scissors and cut-set-cautery, it was so tightly embedded into pannus. Therefore, it had to be divided into several segments for complete removal. Subsequently, a new mechanical valve (St. Jude Medical valve 27 mm; Abbott Laboratories, IL, USA) was placed in supra-annular position. The left atrium was closed, and the patient was weaned off CPB (*Video 3*). Transoesophageal echocardiogram demonstrated no paravalvular leakage.

Aortic cross-clamp, cardiopulmonary bypass, and operation times were 152, 232, and 295 min, respectively. She was extubated 19 h after the operation. She had an uneventful recovery and went home on a postoperative Day 8, being asymptomatic under optimal medical therapy 1 year after surgery. Her echocardiogram showed normal functions of the SAVV and the systemic ventricle.

## Comment

In this patient, anatomical repair (double switch surgery) was not usually indicated for her age. When ccTGA is first diagnosed in adulthood, timely intervention to SAVV is important before deterioration of anatomical right ventricular function.^2^ Once its function has deteriorated due to SAVV regurgitation, it is difficult to return to its baseline even if surgery is performed at that time.^3^ Whereas good long-term outcomes of SAVV replacement in ccTGA patients were reported,^4^ emphasizing the importance of timely valve replacement to achieve the best outcomes.

Exposure of SAVV through median sternotomy is not necessarily easy in ccTGA because the ventricular apex is deviated to the midline, and the valve is directed towards ventral side. Left thoracotomy, as this patient underwent in index surgery, could be an option. Our experience indicated endoscopic approach through the right chest, as usual endoscopic mitral valve surgery, could be another good option to access SAVV in ccTGA with SLL (situs solitus, L-loop, L-malposition) anatomy. Venting of the main pulmonary artery, which lies on the right side of the aorta, played an important role in exposing the ascending aorta. In recent years, trans-catheter approach is also a possible option,^5^ but we report that endoscopic surgery through the right chest could be a versatile option for intervention of SAVV in ccTGA.

## Data Availability

The data underlying this article are available in the article. Any additional readers’ inquiries relating to this article will be shared on reasonable request to the corresponding author.

## Lead author biography

Riku Kato has served as a Cardiovascular Surgery Fellow at Japanese Red Cross Aichi Medical Center Nagoya Daiichi Hospital since 2016. He has a specialist license of cardiovascular surgeon. With involvement in over 500 cases of minimally invasive cardiovascular surgery, he has gained extensive experience in this specialized field. His commitment to learning spans across all areas of cardiovascular surgery.
